# Low T3 syndrome is a strong predictor of poor outcomes in patients with community-acquired pneumonia

**DOI:** 10.1038/srep22271

**Published:** 2016-03-01

**Authors:** Jinliang Liu, Xuejie Wu, Fang Lu, Lifang Zhao, Lingxian Shi, Feng Xu

**Affiliations:** 1 Department of Infectious Diseases, Second Affiliated Hospital, Zhejiang University School of Medicine, Hangzhou, China; 2 Department of Respiratory Medicine, Quzhou People’s Hospital, Quzhou, China

## Abstract

Low T3 syndrome was previously reported to be linked to poor clinical outcomes in critically ill patients. The aim of this study was to evaluate the predictive power of low T3 syndrome for clinical outcomes in patients with community-acquired pneumonia (CAP). Data for 503 patients were analyzed retrospectively, and the primary end point was 30-day mortality. The intensive care unit (ICU) admission rate and 30-day mortality were 8.3% and 6.4% respectively. The prevalence of low T3 syndrome differed significantly between survivors and nonsurvivors (29.1% *vs* 71.9%, *P* < 0.001), and low T3 syndrome was associated with a remarkable increased risk of 30-day mortality and ICU admission in patients with severe CAP. Multivariate logistic regression analysis produced an odds ratio of 2.96 (95% CI 1.14–7.76, *P* = 0.025) for 30-day mortality in CAP patients with low T3 syndrome. Survival analysis revealed that the survival rate among CAP patients with low T3 syndrome was lower than that in the control group (*P* < 0.01). Adding low T3 syndrome to the PSI and CURB-65 significantly increased the areas under the ROC curves for predicting ICU admission and 30-day mortality. In conclusion, low T3 syndrome is an independent risk factor for 30-day mortality in CAP patients.

Community-acquired pneumonia (CAP) is one of the most common and most fatal infectious diseases worldwide^1^, with overall mortality rates of 13% among hospitalized patients and up to 35% in severe CAP patients^2^. It is important for physicians to identify patients with severe pneumonia as early as possible^3^, because rapid assessment of CAP severity and outcome prediction can support appropriate treatments and thereby reduce mortality^4^,^5^,^6^.

To date, many severity scores and biomarkers have been developed for predicting mortality and identifying CAP patients at high risk of mortality^7^. The Pneumonia Severity Index (PSI) and CURB-65 (Confusion, Urea, Respiratory rate, Blood pressure, and age ≥65) are two of the most powerful prediction tools for predicting pneumonia mortality^8^,^9^. The PSI stratifies patients into five risk classes according to twenty criteria, and its ability to predict mortality has been validated in multiple studies^6^,^10^. However, its complexity limits its practicability in emergency situations^10^. In contrast, the CURB-65 categorizes patients into three risk classes using only five easily measurable factors. However, the CURB-65 has not been as extensively studied as the PSI, especially with prospective validation in various patient populations^11^. Moreover, both scores perform poorly in predicting intensive care unit (ICU) admission^12^.

Low T3 syndrome, which is also known as non-thyroidal illness syndrome or sick euthyroid syndrome, is characterized by a low serum level of free triiodothyronine (FT3) accompanied by normal-to-low serum free thyroxine (FT4) and thyroid-stimulating hormone (TSH) levels^13^. Several studies have confirmed that low T3 syndrome is a strong predictor of poor prognosis in critically ill hospitalized patients, including those who have experienced surgery, multiple trauma, respiratory failure, septic shock, cerebrovascular diseases, cardiovascular diseases and severe burns^14^,^15^,^16^,^17^,^18^,^19^,^20^. However, no conclusive data regarding the association between low T3 syndrome and outcomes of CAP are available. The aim of our study was to investigate the predictive value of low T3 syndrome for outcomes in CAP patients.

## Results

### Patient characteristics

A total of 1640 patients hospitalized with pneumonia derived from the community were evaluated in this retrospective study. Eighty-two patients who had a history of thyroid diseases and 114 patients receiving immunosuppressant therapy or presenting with human immunodeficiency virus (HIV) infection were excluded. Another 941 patients who did not complete a serum thyroid hormone test also were excluded. Finally, a total of 503 hospitalized CAP patients were eligible for analysis in this retrospective observational study. The demographic characteristics of these patients are presented in Table 1. The mean age was 63 ± 18 years, and 275 patients (54.7%) were male. Overall, 193 (38.4%) of 503 cases were accompanied by one or more coexisting illnesses, including congestive heart failure (CHF) (12.9%), chronic obstructive pulmonary disease (COPD) (8.5%), chronic renal disease (5.6%), chronic liver disease (2.4%), cerebrovascular disease (7.8%), malignant tumor (4.2%), diabetes mellitus (9.1%), and septic shock (6.4%). In addition, 160 (31.8%) patients had low T3 syndrome. The 30-day mortality and ICU admission rate in the included population were 6.4% and 8.3%, respectively. The median length of stay (LOS) was 11 days (interquartile range [IQR], 7–16 days; Table 1). To exclude potential selection bias, we analyzed the baseline characteristics of patients who did or did not participate in a thyroid hormone test. We found that neither the 30-day mortality nor ICU admission rate showed any significant difference between two groups (Supplemental Table S1).

### Univariate and multivariate analysis of risk factors for ICU admission and 30-day mortality

Compared to those who survived beyond 30 days, non-survivors were older; more likely to also have CHF, COPD, hypoalbuminemia, thrombocytopenia, hyponatremia, low T3 syndrome, and septic shock; and showed a higher C reactive protein (CRP) level, a higher blood urea nitrogen (BUN) level, lower arterial pH, and higher prevalence of severe CAP (SCAP, PSI risk class ≥ IV or CURB-65 score ≥2 or eligible for SCAP criteria; *P* < 0.05). Non-survivors showed significantly lower FT3, FT4 and TSH levels than those who survived (*P* < 0.05; Table 1). A total of 12 risk factors, including 10 variables showing statistical significance in the univariate analysis (*P* < 0.05) as well as both respiratory rate and pulse rate considered to be clinically important (*P* = 0.107 and *P* =0.151, respectively, by Fisher’s exact test), were further analyzed by multivariate logistic regression (Table 2). The results showed that age, CHF, low T3 syndrome, platelet count <10^6^/μl and serum sodium <130 mmol/l are independent risk factors for 30-day mortality. Also, CHF, COPD, pulse >125/min, albumin (ALB) <30 g/dl, and platelet count <10^6^/μl are independent risk factors for ICU admission.

### Low T3 syndrome is associated with increased 30-day mortality

**Figure 1:**
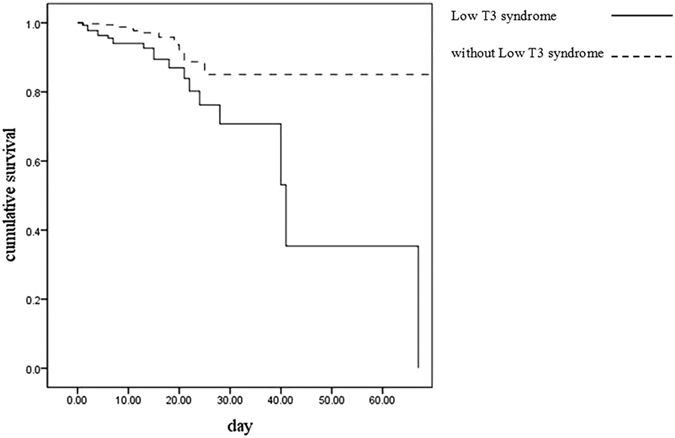
Survival analysis for patients with or without low T3 syndrome.

The characteristics of CAP patients with or without low T3 syndrome are listed in Table 3. CAP patients with low T3 syndrome had a higher 30-day mortality (odds ratio [OR] = 6.23, 95% confidence interval (CI) 2.81–13.81) and higher rate of ICU admission (OR = 3.97, 95% confidence interval [CI] 2.06–7.63). Most importantly, we found that among severe CAP patients, low T3 syndrome was associated with a remarkably increased risk for 30-day mortality (OR = 12.52, 95% CI 2.77–56.62 for the PSI risk class ≥ V group, OR = 9.75, 95% CI 2.12–44.76 for the CURB-65 score ≥2 group, and OR = 6.96, 95% CI 1.94–24.94 for SCAP, respectively, *P* < 0.05) and ICU admission (OR = 5.12, 95% CI 1.78–14.74 for the PSI risk class ≥ IV, OR = 3.96, 95%CI 1.45–10.77 for the CURB-65 score ≥2 group, and OR = 2.29, 95% CI 0.95–5.49 for SCAP, respectively, *P* < 0.05). Consistently, the survival analysis revealed that CAP patients with low T3 syndrome showed a lower survival rate than the patients without low T3 syndrome (*P* = 0.003 by Log-rank test, Fig. 1).

### Correlation between FT3, FT4, and TSH levels and outcomes of CAP patients

**Figure 2:**
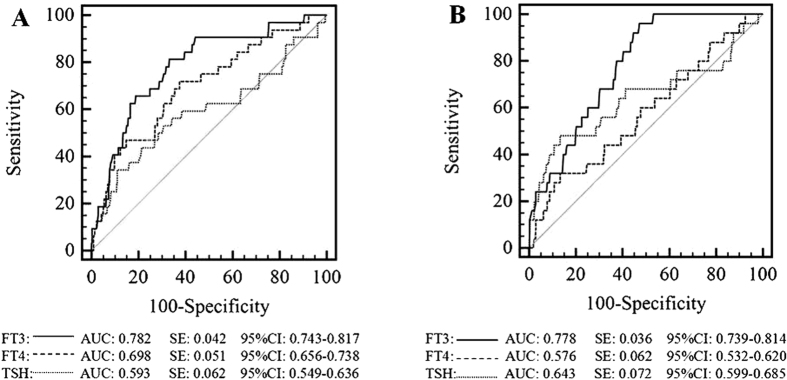
ROC curves for FT3, FT4, and TSH for 30-day mortality (**A**) and ICU admission (**B**).

**Figure 3:**
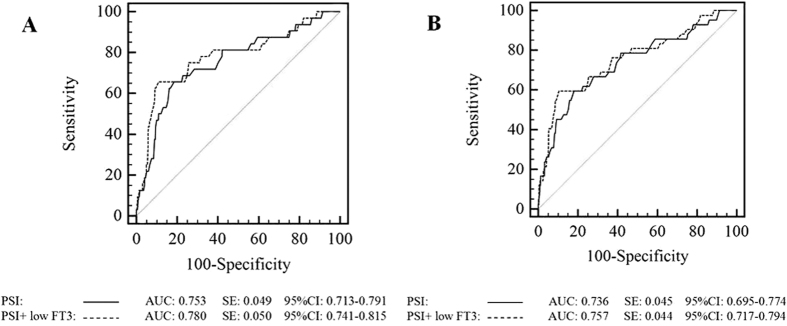
ROC curves for PSI and low FT3 + PSI for 30-day mortality (**A**) and ICU admission (**B**).

**Figure 4:**
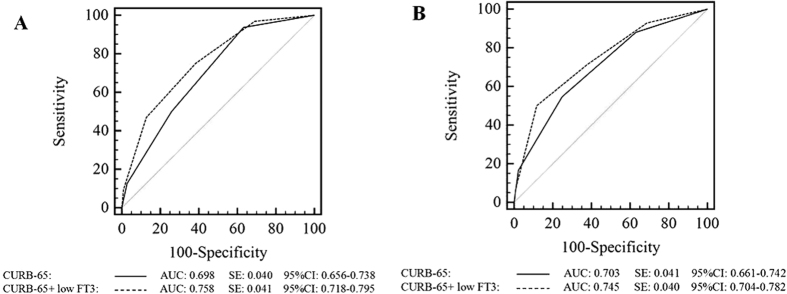
ROC curves for CURB-65 and low FT3 + CURB-65 for 30-day mortality (**A**) and ICU admission (**B**).

Because low T3 syndrome was found to be an independent risk factor for 30-day mortality in the present study, we performed a receiver operating characteristic (ROC) curve analysis of the ability of FT3, FT4, and TSH levels to predict the 30-day mortality and ICU admission among CAP patients (Fig. 2). We found that the areas under the ROC curves (AUCs) for FT3 were 0.782 (95% CI 0.743–0.817) for predicting 30-day mortality and 0.778 (95% CI 0.739–0.814) for predicting ICU admission, and these values were superior to those for serum FT4 and TSH. The best FT3 cut-off points for predicting 30-day mortality and ICU admission were FT3 < 3.06 pmol/l and FT3 < 3.21 pmol/l, respectively. Next, we made low T3 syndrome as an additional 20 points to the PSI and 1 point to CURB-65 to create two new scores and compared their predictive abilities with those of the PSI and CURB-65 alone for 30-day mortality and ICU admission (Figs 3 and 4). Interestingly, both low FT3 + PSI and low FT3 + CURB-65 had significantly increased AUCs for predicting 30-day mortality, compared to the individual indices (0.780 *vs* 0.753, *P* = 0.035 and 0.758 *vs* 0.698, *P* = 0.003, respectively). In addition, low FT3 + CURB-65 had a significantly greater AUC for predicting ICU admission than CURB-65 alone (0.745 *vs* 0.703, *P* = 0.021). However, low FT3 + PSI did not show great accuracy than PSI alone for predicting ICU admission (0.757 *vs* 0.736, *P* = 0.084).

## Discussion

CAP is one of the most common diseases requiring hospital admission and a major cause of death worldwide. It is important to identify patients with severe pneumonia and those who have the worst prognosis in order to provide the most appropriate treatments. Low T3 syndrome was reported to be highly prevalent in critically ill patients and has been proven to be a predictor of poor outcome in these patients^21^,^22^,^23^. Our study showed that low T3 syndrome is an independent risk factor for 30-day mortality among CAP patients, and the survival curves confirmed that CAP patients with low T3 syndrome had a lower survival rate than those without low T3 syndrome. We also found that the serum FT3 level offers better diagnostic accuracy than FT4 and TSH levels, and adding low FT3 criteria to the PSI or CURB-65 scores could significantly increased the AUCs for predicting 30-day mortality and ICU admission, compared to those for the individual scores.

Low T3 syndrome has been identified as a useful adaptation in the negative feedback regulation to preserve energy when patients suffer from an acute critical illness^24^. However, it remains controversial whether low T3 syndrome represents a protective or a maladaptive response to prolonged critical illness^25^. During acute illness, the occurrence of low T3 syndrome can be explained by changes in thyroid hormone binding, peripheral thyroid hormone uptake and the expression and activity of the type-1 deiodinases (D1) and type-3 deiodinases (D3)^26^. As patients with acute critical illnesses usually experience concomitant fasting, the decreased thyroid hormone availability may reflect an adaptive attempt to reduce energy expenditure and, thus, appears to be beneficial^27^. In addition, the increased D3 activity could optimize the bacterial killing capacity of neutrophilic granulocytes^28^.

Unlike in the acute phase, the thyroid axis appears to be regulated by different mechanisms during prolonged critical illness. Low T3 syndrome in these patients is found to result from the low expression of the thyrotropin-releasing hormone (TRH) gene in the paraventricular nucleus (PVN), which impairs the hypothalamic stimulation of the thyrotropes^29^. Peripheral tissues adapt to the decreased thyroid hormones status by increasing levels of thyroid hormone transporters, local activation of thyroid hormone and gene expression of the active receptor isoforms^30^,^31^,^32^,^33^. Different from acute critical illness, the low T3 level correlates with markers of muscle breakdown and of bone loss, which indicate a hypercatabolic maladaptive response in prolonged critical illness^34^. In summary, low T3 syndrome during prolonged critical illness appears to differ from changes observed during acute critical illnesses, both in its mechanisms and in its influence on prognosis.

In the present study, low T3 syndrome was identified as an independent risk factor associated with poor 30-day mortality among CAP patients. We found that the magnitude of the decrease in the serum FT3 levels was correlated with the severity of CAP, and serum FT3 shows accurate prognostic value for predicting 30-day mortality and ICU admission among CAP patients. This may be attributed to large amounts of inflammatory cytokines, including tumor necrosis factor (TNF)-α, interleukin (IL)-1 and IL-6, which are present during critical pneumonia. These cytokines are considered to be putative mediators of low T3 syndrome^35^,^36^. Additionally, D3, which is viewed as the major thyroid hormone inactivating enzyme, was shown to be highly expressed in infiltrating neutrophilic granulocytes during bacterial infections^28^, which may lead to low T3 levels during an acute infection.

The PSI and CURB-65 scores have shown good diagnostic accuracy for predicting mortality among pneumonia patients in previous studies^8^,^9^, but limited accuracy for predicting ICU admission. Although the low serum FT3 was not included as a variable in any previous severity scores for CAP, low FT3 criteria indeed significantly improved the performance of PSI and CURB-65 for predicting 30-day mortality and ICU admission in CAP patients.

There are a few limitations in this study. First, this was a retrospective single-center study. Second, the result of endocrine-based testing may be related to the timing of sampling. It has been reported that serum thyroid hormone level may require 4 days to reach a nadir after the onset of critical illness^37^. In addition, dopamine, which can induce iatrogenic hypothyroidism in patients with critical illness^38^,^39^, was not excluded as a confounder in our study.

In conclusion, we found that low T3 syndrome is a reliable predictor of worse outcomes in hospitalized CAP patients. The serum FT3 level showed great performance for prediction of 30-day mortality and ICU admission in CAP patients, and was found to be even more diagnostically accurate than the PSI and CURB-65. The finding of the present study should be conformed in multicenter large prospective studies, and future studies are needed to elucidate the underlying mechanisms by which low T3 syndrome affects CAP patients.

## Materials and Methods

### Study population

We retrospectively evaluated the records of all hospitalized patients who were diagnosed with CAP between January 2010 and December 2013 at the Second Affiliated Hospital, Zhejiang University School of Medicine. According to the American Thoracic Society (ATS)/Infectious Disease of Society of America (IDSA) guidelines, the diagnosis of CAP is based on the presence of select clinical features (e.g., cough, fever, sputum production, and pleuritic chest pain) and is supported by imaging of the lung, usually by chest radiography, with or without supporting microbiological data^11^,^40^. Eligible patients were older than 14 years, and had available serum thyroid hormone levels. Patients were excluded if they had an overt history of hypothyroidism or hyperthyroidism diagnosed during admission, or also presented with HIV infection, had undergone organ transplantation, or were receiving immunosuppressant therapy (>20 mg/day of prednisone or equivalent). The Ethics Committee at the Second Affiliated Hospital, Zhejiang University School of Medicine approved this study. All aspects of the study were performed in accordance with relevant guidelines and regulations^41^.

### Data collection and definition of severe CAP

Baseline clinical information was obtained for each patient, including age, gender, comorbid diseases such as CHF, COPD, chronic renal diseases, chronic liver diseases, cerebrovascular disease, malignancy, diabetes mellitus, antimicrobial pre-treatment, physical examination results, septic shock (severe sepsis plus hypotension that was not reversed with fluid resuscitation), laboratory data such as white blood cell (WBC) count and platelet count, and levels of CRP, ALB, BUN, glucose, serum sodium, FT3, FT4, and TSH, and radiologic findings (new or progressive pulmonary infiltrate) within 24 h after admission. To evaluate the severity of CAP, the PSI, CURB-65 and SCAP scores were independently calculated^8^,^9^,^42^,^43^. The thyroid hormone levels were measured on the next morning after admission using commercial radioimmunoassay kits (Abbott Architect i2000 analyzer). According to the manufacturer’s instructions, the normal reference intervals used were: 3.1–7.5 pmol/l for FT3, 8.9–20.6 pmol/l for FT4, and 0.35–4.6 mIU/l for TSH. In accordance with the manufacturer’s standard value, low T3 syndrome was defined by a low serum FT3 level (<3.1 pmol/l) with low or normal serum FT4 and TSH levels (≤20.6 pmol/l and ≤4.6 mIU/l, respectively), and patients who had been diagnosed with hypothyroidism, hyperthyroidism, or thyroiditis were excluded^13^,^44^. The primary end point was 30-day mortality, which was defined as death due to any cause within 30 days from hospital admission. Secondary end points were LOS in the hospital and ICU admission.

### Statistical analysis

All continuous variables are presented as mean ± standard deviation or as median (IQR). Categorical variables are presented as a number (%). Comparisons between continuous variables were performed using the unpaired t-test. Qualitative/categorical variables were compared between groups using the chi-square test or Fisher’s exact test. Univariate analysis and multiple logistic regression analysis were performed to determine independent risk factors for 30-day mortality and ICU admission. The ORs and corresponding 95% CI were calculated for all variables. The ROC curves for the ability of FT3, FT4 and TSH to predict the 30-day mortality and ICU admission were analyzed. The AUCs were compared using Delong-Delong nonparametric method. Kaplan-Meier analysis was used to estimate the probability of survival and comparison was made by Log-rank test. The best FT3 cut-off points for predicting 30-day mortality and ICU admission were determined by the Youden index. A *P* value of <0.05 was considered statistically significant. The results were analyzed using SPSS version 18.0 for Windows (SPSS Inc., Chicago, IL, USA).

## Additional Information

**How to cite this article**: Liu, J. *et al.* Low T3 syndrome is a strong predictor of poor outcomes in patients with community-acquired pneumonia. *Sci. Rep.* **6**, 22271; doi: 10.1038/srep22271 (2016).

## Supplementary Material

Supplementary Information

## Figures and Tables

**Table 1:** Baseline characteristics of survivors and non-survivors among CAP patients.

	Total	Survivor	Non-survivor	*P* value
Patients, N (%)	503 (100%)	471 (93.6%)	32 (6.4%)	—
LOS, median (IQR)	11 (7–16)	11 (8–15)	14 (6.25–21.75)	0.354^#^
Age, mean (±SD), y	63 ± 18	63 ± 18	74 ± 16	0.001^#^
Male sex, N (%)	275 (54.7%)	256 (54.4%)	19 (59.4%)	0.581
Antimicrobial pre-treatment, N (%)	175 (34.8%)	160 (34.0%)	15 (46.9%)	0.138
Congestive heart failure, N (%)	65 (12.9%)	53 (11.3%)	12 (37.5%)	<0.001
COPD, N (%)	43 (8.5%)	36 (7.6%)	7 (21.9%)	0.005
Chronic renal diseases, N (%)	28 (5.6%)	26 (5.5%)	2 (6.3%)	0.862
Chronic liver diseases, N (%)	12 (2.4%)	11 (2.3%)	1 (3.1%)	0.550^*^
Cerebrovascular diseases, N (%)	39 (7.8%)	35 (7.4%)	4 (12.5%)	0.299
Malignancy, N (%)	21 (4.2%)	20 (4.2%)	1 (3.1%)	0.759
Diabetes mellitus, N (%)	46 (9.1%)	45(9.6%)	1 (3.1%)	0.837
Septic shock, N (%)	32 (6.4%)	26 (5.5%)	6 (18.8%)	0.038
Pulse rates ≥125/min, N (%)	11 (2.2%)	9 (1.9%)	2 (6.3%)	0.151^*^
Respiratory rates ≥30/min, N (%)	9 (1.8%)	7 (1.5%)	2 (6.3%)	0.107^*^
Low T3 syndrome, N (%)	160 (31.8%)	137 (29.1%)	23 (71.9%)	<0.001
FT3, pmol/l	3.43 ± 0.98	3.50 ± 0.95	2.47 ± 0.92	<0.001^#^
FT4, pmol/l	14.76 ± 2.93	14.89 ± 2.89	12.85 ± 2.89	<0.001^#^
TSH, mIU/l	1.6 ± 1.0	1.64 ± 1.07	1.22 ± 1.04	0.041^#^
ALB <30 g/dl, N (%)	115 (22.7%)	98 (20.8%)	17 (53.1%)	<0.001
WBC count <4 or >10 × 10^9^/L	156 (31.0%)	143 (30.4%)	13 (40.6%)	0.224
CRP >150 mg/dl, N (%)	90 (17.9%)	80 (17.0%)	10 (31.3%)	0.042
BUN >7 mmol/l, N (%)	151 (30.0%)	135 (28.7%)	16(50%)	0.011
Platelet count <10^6^/ul, N (%)	81 (16.1%)	66 (14.0%)	17 (53.1%)	<0.001
Arterial PH <7.35, N (%)	27 (5.4%)	22 (4.7%)	5 (15.6%)	0.008
Glucose ≥ 250 mg/dl, N (%)	21 (4.2%)	19 (4.0%)	2 (6.3%)	0.544
Hematocrit <30%, N (%)	104 (20.7%)	94 (20.0%)	10 (31.3%)	0.127
Serum sodium <130 mmol/l, N (%)	27 (5.4%)	20 (4.2%)	7 (21.9%)	<0.001
Multilobar pneumonia, N (%)	141 (28.0%)	129 (27.4%)	12 (37.5%)	0.218
CURB-65 ≥ 2	138 (27.4%)	122 (25.9%)	16 (50.0%)	0.003
PSI class ≥ IV	123 (24.5%)	102 (21.7%)	21 (65.6%)	<0.001
SCAP	147 (29.2%)	127 (27.0%)	20 (62.5%)	<0.001
ICU admission, N (%)	42 (8.3%)	11 (2.3%)	31 (96.9%)	<0.001
30-day mortality, N (%)	32 (6.4%)	—	—	—

^#^*P* values and *P values were calculated by unpaired t-test or Fisher exact test, respectively. Other *P* values were performed by chi-square test.

**Table 2:** Multivariate logistic regression analyses of risk factors associated with 30-day mortality and ICU admission among CAP patients.

	30-day mortality			ICU admission		
	OR	95% CI	*P*	OR	95% CI	*P*
Age, year	1.03	1.00–1.06	0.045	1.02	0.99–1.04	0.178
CHF	5.02	1.86–13.54	0.001	3.89	1.61–9.40	0.003
COPD	2.59	0.75–8.95	0.133	3.72	1.28–10.77	0.015
Septic shock	1.23	0.39–3.93	0.724	1.66	0.61–4.53	0.327
Pulse >125/min	2.69	0.37–19.66	0.331	6.04	1.22–29.78	0.027
Respiratory rate	3.51	0.43–28.68	0.241	4.38	0.71–26.82	0.111
low T3 syndrome	2.96	1.14–7.76	0.025	1.76	0.78–3.97	0.175
ALB <30 g/dl	2.34	0.96–5.72	0.061	2.62	1.18–5.82	0.018
CRP >150 mg/dl	1.64	0.63–4.27	0.310	1.23	0.51–2.98	0.640
BUN >7mmol/l	1.62	0.68–3.88	0.277	1.69	0.78–3.65	0.181
Platelet count <10^6^/ul	2.45	1.01–5.92	0.047	2.95	1.34–6.48	0.007
Serum sodium <130 mmol/l	3.72	1.15–12.08	0.029	2.78	0.87–8.71	0.080

**Table 3:** Characteristics of CAP patients with or without low T3 syndrome.

	Total	Low T3 syndrome	without Low T3 syndrome	*P* value
Patients, N (%)	503 (100%)	160 (31.8%)	343 (68.2%)	—
Age, mean (±SD), y	63 ± 18	66 ± 19	62 ± 17	0.010^#^
Male sex, N (%)	275 (54.7%)	74 (54.4%)	201 (54.8%)	0.941
LOS, median (IQR), days	11 (7–16)	13 (9–18)	10 (7–15)	<0.001^#^
ICU admission, N (%)	42 (8.3%)	26 (16.3%)	16 (4.7%)	<0.001
30-day mortality, N (%)	32 (6.4%)	23 (14.4%)	9 (2.6%)	<0.001
PSI class I-III, N (%)	380 (100%)	97 (25.5%)	283 (74.5%)	—
ICU admission, N (%)	17 (4.5%)	6 (6.2%)	11 (3.9%)	0.345
30-day mortality, N (%)	11 (2.9)	4 (4.1%)	7 (2.5%)	0.403
PSI class IV-V, N (%)	123 (100%)	63 (51.2%)	60 (48.8%)	—
ICU admission, N (%)	25 (20.3%)	20 (31.7%)	5 (1.8%)	0.001
30-day mortality, N (%)	21 (17.1%)	19 (30.2%)	2 (3.3%)	<0.001
CURB-65 0-1, N (%)	365 (100%)	95 (26.0%)	270 (74.0%)	—
ICU admission, N (%)	19 (5.2%)	9 (9.5%)	10 (3.7%)	0.029
30-day mortality, N (%)	16 (4.4%)	9 (9.5%)	7 (2.6%)	0.005
CURB-65 ≥ 2, N (%)	138 (100%)	65 (47.1%)	73 (52.9%)	—
ICU admission, N (%)	23 (16.7%)	17 (26.2%)	6 (8.2%)	0.005
30-day mortality, N (%)	16 (11.6%)	14 (21.5%)	2 (2.7%)	0.001
Non-SCAP	356 (100%)	86 (24.2%)	270 (75.8%)	—
ICU admission, N (%)	15 (4.2%)	8 (9.3%)	7 (2.6%)	0.007
30-day mortality, N (%)	12 (3.4%)	6 (7.0%)	6 (2.2%)	0.033
SCAP	147 (100%)	74 (50.3%)	73 (49.7%)	—
ICU admission, N (%)	27 (18.4%)	18 (24.3%)	9 (12.3%)	0.060
30-day mortality, N (%)	20 (13.6%)	17 (23.0%)	3 (4.1%)	0.001

^#^*P* values were calculated by unpaired t-test. Other *P* values were performed by chi-square test.
