# Supplementary material for: Low T3 syndrome is a strong predictor of poor outcomes in patients with community-acquired pneumonia
Source: Sci Rep. 2016 Mar 1;6:22271. doi: 10.1038/srep22271 (PMC4772089; doi:10.1038/srep22271)
Supplement: Supplementary Information [file srep22271-s1.pdf]

Low T3 syndrome is a strong predictor of poor outcomes in patients with community-acquired pneumonia

Jinliang Liu, Xuejie Wu, Fang Lu, Lifang Zhao, Lingxian Shi, Feng Xu

Table S1: Baseline characteristics of CAP patients who did or did not complete the thyroid hormone tests. Most *P* values were calculated by chi-square test. #*P* values were calculated by unpaired t-test.

	Patients who did not complete a thyroid hormone test	Patients who complete a thyroid hormone test	*P* value
Number, N	941	503	-
Age, mean (±SD), y	63 ± 19	63 ± 18	0.997#
Male Sex, N (%)	582 (61.8%)	275 (54.7%)	0.008
PSI IV-V	318 (33.8%)	123 (24.5%)	<0.001
CURB-65 2-5	239 (25.4%)	138 (27.4%)	0.401
SCAP	217 (23.1%)	147 (29.2%)	0.010
30-day mortality N, %	82 (8.7%)	32 (6.4%)	0.114
ICU admission N, %	107 (11.3%)	42 (8.3%)	0.072
